# Effectiveness of Fatigue-Reducing Interventions in Pediatric Rheumatic Diseases: A Systematic Review

**DOI:** 10.7759/cureus.83056

**Published:** 2025-04-27

**Authors:** Afra Mohamed Awad Abdu Alla, Ashgan Elfadel Eltyeeb Elnour, Aminat Olayiwola, Ehab Zahran, Rayan Zakria Mohamed Edris, Nazik Abbas Mohammed Ahmmed

**Affiliations:** 1 Department of Pediatrics, Tuwaiq Medical Complex, Riyadh, SAU; 2 Department of Pediatric Hematology and Oncology, King Saud Medical City, Riyadh, SAU; 3 Department of Pediatrics, The Royal London Hospital, London, GBR; 4 Department of Pediatrics, Cambridge University Hospitals NHS Foundation Trust, Cambridge, GBR; 5 Department of Pediatrics, Leeds General Infirmary Hospitals, Leeds, GBR; 6 Department of Pediatrics, Ateika Medical Health Center - Riyadh First Health Cluster, Riyadh, SAU

**Keywords:** adolescents, children, fatigue, interventions, pediatrics

## Abstract

There are few intervention trials aimed at lowering fatigue in pediatric rheumatic conditions (PRCs), despite the fact that it is a common and upsetting symptom in these patients. The study's primary goal is to thoroughly examine the data pertaining to the effectiveness of treatments meant to lessen fatigue in PRC patients. We followed the Preferred Reporting Items for Systematic Reviews and Meta-Analyses (PRISMA) guidelines to search for relevant studies across four different databases (PubMed, Web of Science, Scopus, and Google Scholar). A total of 493 records were identified through database searches, and after removing 196 duplicates, 297 unique studies remained for screening. Following title screening and eligibility assessment, 45 studies were excluded for various reasons, and 10 studies met the inclusion criteria for this systematic review. The interventions included exercise treatment on land and in water, prednisolone, vitamin D and creatine supplements, psychological counseling, and a program for transitioning into a mature rheumatology program. Every included study measured fatigue using self-reported questionnaires. Two randomized controlled studies found land-based exercise treatment to be ineffective, while one pre-post intervention research found it to be useful. Compared to land-based exercise therapy, aquatic-based physical therapy was found to be more beneficial. Prednisolone combined with vitamin D significantly reduced subjective fatigue in two placebo-controlled trials. Creatine did not seem to be beneficial. The effectiveness of the present therapies to lessen fatigue in PRCs is not sufficiently supported by the available data. Future research should focus on intervention studies targeted at treating fatigue in adolescents and children with PRCs, as indicated by the small number of investigations, non-comparable therapies, risk of bias, and unclear outcomes of the included studies. It is necessary to identify potential underlying biological and psychological pathways as potential therapy targets in order to lessen fatigue symptoms in kids and teenagers with PRCs.

## Introduction and background

Fatigue is one of the most prevalent and debilitating symptoms experienced by children and adolescents with pediatric rheumatic diseases (PRDs), significantly affecting their daily functioning, quality of life, and overall well-being [1]. PRDs, including juvenile idiopathic arthritis (JIA), systemic lupus erythematosus (SLE), juvenile dermatomyositis (JDM), and other inflammatory conditions, are characterized by chronic inflammation, pain, and disability, which contribute to persistent fatigue [2]. Unlike transient tiredness, fatigue in PRDs is often severe, unrelieved by rest, and multifactorial in origin, arising from a combination of disease activity, inflammation, medication side effects, sleep disturbances, and psychosocial factors [3]. However, fatigue is particularly challenging to treat and measure due to its subjective nature, fluctuating intensity, and the lack of standardized assessment tools. Despite its widespread impact, fatigue remains an underrecognized and undertreated symptom in pediatric rheumatology, necessitating a systematic evaluation of interventions aimed at its management [4].

Various interventions have been proposed to reduce fatigue in pediatric patients with PRDs, ranging from pharmacological treatments to non-pharmacological approaches such as physical activity programs, cognitive-behavioral therapy (CBT), sleep hygiene strategies, and dietary modifications [5]. Currently available treatments are limited in number and effectiveness; pharmacological interventions often aim to reduce disease activity, while non-pharmacological approaches focus on improving physical conditioning, mental health, and sleep quality. Pharmacological interventions often include anti-inflammatory or immunosuppressive therapies, which may indirectly alleviate fatigue by controlling disease activity. However, concerns regarding long-term medication use, potential side effects, and variable patient responses highlight the importance of non-pharmacological strategies. Exercise programs, including aerobic training and strength-building exercises, have been shown to improve endurance, reduce pain, and enhance overall energy levels [6]. Similarly, psychological interventions, such as CBT, aim to address maladaptive coping mechanisms, depression, and anxiety, which are frequently associated with fatigue in PRDs. Given the multifactorial nature of fatigue, a multidisciplinary approach incorporating medical, psychological, and lifestyle interventions may offer the most effective management strategy [7].

Despite increasing research on fatigue-reducing interventions in pediatric rheumatology, the effectiveness of various strategies remains uncertain due to heterogeneous study designs, small sample sizes, and variations in outcome measures. This inconsistency further complicates the development of clear treatment protocols and standardized clinical recommendations. Current clinical guidelines provide limited recommendations on fatigue management, emphasizing the need for a comprehensive synthesis of available evidence to inform clinical practice [8]. This systematic review aims to critically evaluate and synthesize the existing literature on the effectiveness of interventions targeting fatigue in pediatric patients with PRDs. By identifying the most effective strategies, highlighting gaps in knowledge, and providing evidence-based recommendations, this review seeks to support healthcare providers in optimizing fatigue management and improving the quality of life for children and adolescents with PRDs.

## Review

Methodology

Study Design

This systematic review was conducted following the Preferred Reporting Items for Systematic Reviews and Meta-Analyses (PRISMA) guidelines to ensure methodological rigor and transparency [9]. A predefined protocol guided the identification, selection, data extraction, and synthesis of relevant studies examining the effectiveness of fatigue-reducing interventions in PRDs.

Eligibility Criteria

Studies were included if they investigated fatigue-reducing interventions in pediatric patients (aged 18 years or younger) diagnosed with JIA, SLE, JDM, or other inflammatory conditions. Only studies that assessed the effectiveness of interventions using validated fatigue measurement tools and were published in peer-reviewed journals in English were considered. Studies involving adult populations, those without clear fatigue assessment as an outcome, and review articles, case reports, editorials, or conference abstracts without full-text availability were excluded.

Search Strategy

A comprehensive literature search was conducted across PubMed, Web of Science, Scopus, and Google Scholar to identify relevant studies published from inception to the present. The search strategy incorporated Medical Subject Headings (MeSH) terms and free-text keywords related to PRDs, fatigue, and interventions, using Boolean operators to refine search results. The detailed search strategy is provided in Table 1.

**Table 1:** Search Engines and Strategy to Look for Relevant Studies

Search Engine	Search Strategy
PubMed	("Fatigue"(MeSH) OR fatigue) AND (pediatric OR children OR adolescents) AND ("Rheumatic Diseases"(MeSH) OR "Juvenile Idiopathic Arthritis" OR "Systemic Lupus Erythematosus") AND (intervention OR therapy OR treatment OR rehabilitation) AND (effectiveness OR outcome).
Web of Science	TS=(fatigue AND (pediatric OR children OR adolescents) AND ("rheumatic diseases" OR "juvenile idiopathic arthritis" OR "systemic lupus erythematosus") AND (intervention OR therapy OR treatment) AND (effectiveness OR outcome)).
Scopus	TITLE-ABS-KEY(fatigue AND (pediatric OR children OR adolescents) AND ("rheumatic diseases" OR "juvenile idiopathic arthritis" OR "systemic lupus erythematosus") AND (intervention OR treatment OR therapy) AND (effectiveness OR outcome)).
Google Scholar	"Fatigue reduction" AND (pediatric OR children OR adolescents) AND ("rheumatic diseases" OR "juvenile idiopathic arthritis" OR "systemic lupus erythematosus") AND (intervention OR therapy OR treatment) AND (effectiveness OR outcome).

Study Selection

All identified records were imported into a reference management software, EndNote X9 (Clarivate Analytics, Philadelphia, Pennsylvania), and duplicates were removed. Two independent reviewers screened the titles and abstracts of identified studies to determine eligibility. Full texts of potentially relevant studies were retrieved and assessed against the inclusion and exclusion criteria. Any disagreements between reviewers were resolved through discussion, with a third reviewer consulted when necessary. A PRISMA flow diagram was used to document the study selection process.

Data Extraction

Data extraction was conducted using a standardized form to ensure consistency and completeness. The extracted data included study characteristics (author, year, country, and study design), participant details (age, gender, disease type, and disease duration), and specifics of the intervention (type, duration, frequency, and intensity). Comparator details were also recorded where applicable. Fatigue outcome measures were collected, including the assessment tools used and reported fatigue levels before and after the intervention. Key findings from each study were systematically documented. Two independent reviewers carried out the data extraction, and any discrepancies were resolved through discussion to maintain accuracy and reliability.

Quality Assessment

The methodological quality and risk of bias of the included studies were assessed using the Cochrane Risk of Bias 2 (RoB 2) tool [10]. Each study was evaluated based on criteria such as randomization process, deviations from the intended intervention, missing outcome data, measurement of the outcome, and selective reporting. The assessment was conducted independently by two reviewers, with disagreements resolved through consensus.

Results

Search Results

**Figure 1:**
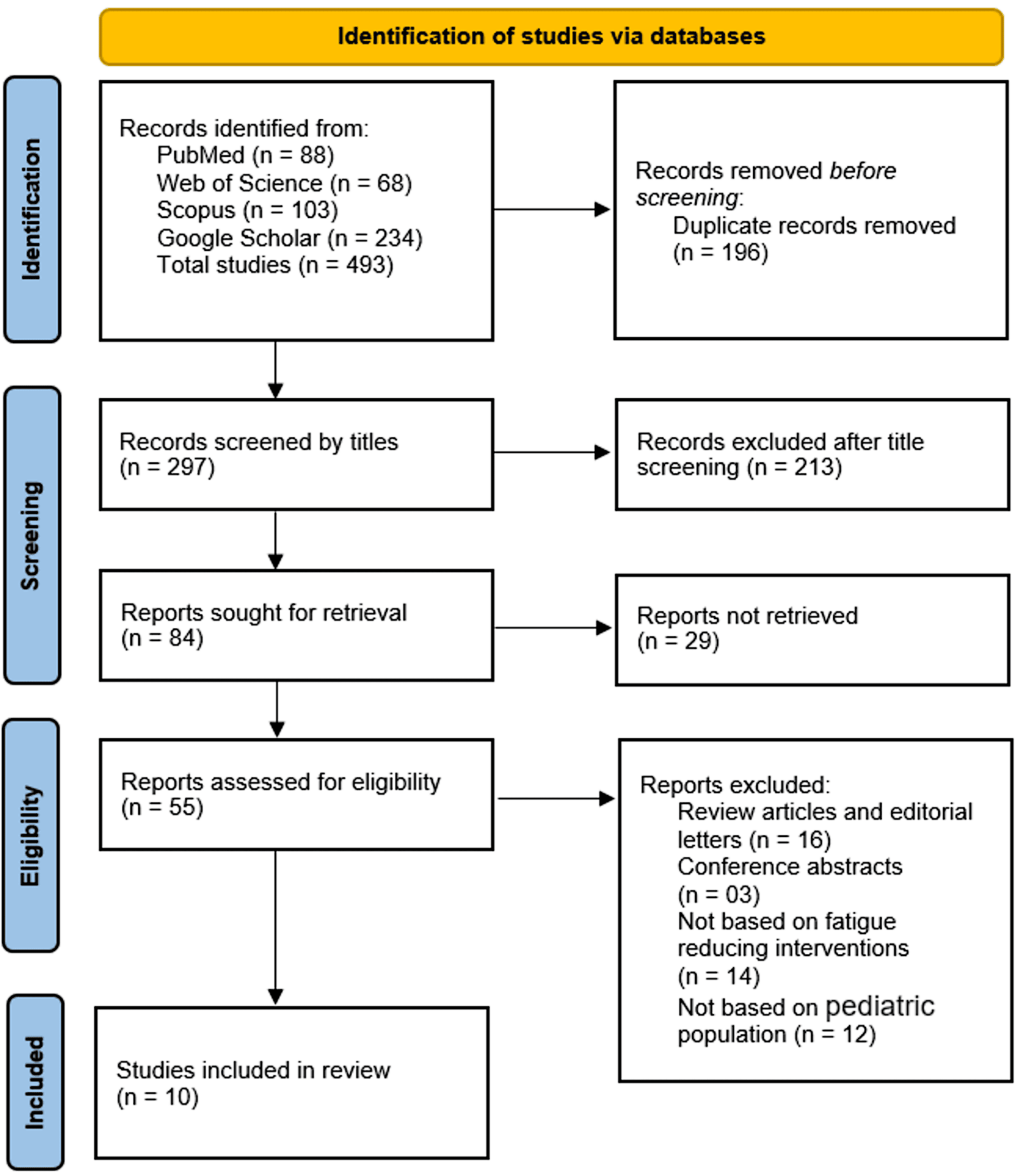
PRISMA Flowchart PRISMA: Preferred Reporting Items for Systematic Reviews and Meta-Analyses

A total of 493 records were identified through database searches, including PubMed (n = 88), Web of Science (n = 68), Scopus (n = 103), and Google Scholar (n = 234). After removing 196 duplicate records, 297 unique studies remained for title screening. Following the initial title screening, 213 records were excluded, leaving 84 reports for further assessment. Among these, 29 reports could not be retrieved, reducing the number of studies assessed for eligibility to 55. After a detailed review, 45 studies were excluded for various reasons: 16 were review articles or editorial letters, three were conference abstracts, 14 did not focus on fatigue-reducing interventions, and 12 did not involve a pediatric population. Ultimately, 10 studies met the inclusion criteria and were included in this systematic review (Figure 1).

Characteristics of Included Studies

The systematic review included 10 studies investigating fatigue-reducing interventions in PRDs. These studies were conducted across various countries, including the Netherlands, Canada, the United States, Egypt, Norway, Brazil, and Belgium. The study designs encompassed randomized controlled trials (RCTs), pre-post intervention studies, crossover trials, controlled trials, and quasi-experimental designs. The sample populations primarily consisted of adolescents and children diagnosed with JDM, JIA, JRA, and SLE (Table 2).

**Table 2:** Key Characteristics of Studies Included in This Systematic Review JDM: Juvenile Dermatomyositis, PedsQL-MFS: Pediatric Quality of Life Inventory Multidimensional Fatigue Scale, RCT: Randomized Controlled Trial, K-FSS: Kinesiology Fatigue Severity Scale, AGIE: Aerobic Group Exercise Intervention, LBE: Low-Impact Aerobic Exercise, JRA: Juvenile Rheumatoid Arthritis, SLE: Systemic Lupus Erythematosus, PROMIS: Patient-Reported Outcomes Measurement Information System, CIS-20: Checklist Individual Strength 20-item, MFI-20: Multidimensional Fatigue Inventory 20-item, ES: Effect Size

Author and Publishing Year	Country	Study Design	Sample Size	Participants	Intervention	Controls	Fatigue Measure	Timing of Fatigue Measurement	Results Mean (95% CI) Mean±SD	Key Findings
Habers et al., (2016) [11]	Netherlands	Multicenter RCT	26	Adolescents with JDM	Individually tailored home-based training (resistance & strength exercises)	Usual care	PedsQL-MFS	Immediately post-intervention	Total score follow-up: IG = 75 (71-79), WCG = 74 (69-78) Δ (95% CI): 1 (-5-7) P=0.8	No statistical difference in fatigue scores
Houghton et al., (2018) [12]	Canada	Pre-post intervention	24	Adolescents with JDM	6-month home-based program (aerobic & resistance exercises)	No control group	PedsQL-MFS	During & after intervention	Total fatigue scores: Baseline = 57.9 ± 18.2, 3 months = 66.0 ± 18.0, 6 months = 69.9 ± 8.9, 12 months = 67.3 ± 17.2, P = 0.034	Fatigue decreased, but not statistically significant at 12 months
Sule and Fontaine, (2019) [13]	United States	Single-center RCT	33	Adolescents with JDM	Resistance exercise training (2 sessions/week for 12 weeks)	The control group performed normal activities	K-FSS	After intervention	Baseline: IG = 5.3 ± 0.8, CG = 4.8 ± 1.4; after intervention: IG = ?, CG = ? No statistical differences between IG and CG	No significant difference between groups
Samhan et al., (2019) [14]	Egypt	Crossover trial	14	JDM patients	AGIE + LBE for 4 weeks, followed by LBE alone	AGIE 45 min, 2x per week	PedsQL-MFS	After intervention	Baseline similar in AQBE and LBE: ±35; AQBE improved to 61.9 ± 3.9; LBE improved to 44.2 ± 2.3	AGIE decreases fatigue
Kvien et al., (1982) [15]	Norway	Parallel blinded RCT	Not reported	Children with JRA	Prednisolone 0.4 mg/kg for 7 days	Placebo tablets	10cm fatigue scale	At 7 and 77 days	Mean (ranges): Prednisolone = 0 (-4-4), Placebo = -1 (-6-0), p = 0.03	Significant reduction in fatigue in the prednisolone group
Lima et al., (2016) [16]	Brazil	RCT	40	Adolescents with SLE	Vitamin D supplementation (50,000 IU/week for 6 months)	Identical placebo tablets	PedsQL-MFS	After intervention	Global score after intervention: VITD = 3.15 ± 1.44, Placebo = 4.30 ± 1.33	Vitamin D supplementation improves disease activity
Dover et al., (2021) [17]	Canada	RCT	13	Children with JDM	Calcium & vitamin D supplementation	Placebo tablets	PedsQL-MFS	7 weeks, post-intervention	Mean difference when on creatine compared to placebo = 3.6, P = 0.982	No effect on fatigue
Cunningham et al., (2019) [18]	United States	Pre-post intervention	14	Adolescents with SLE & JDM	Cognitive-behavioral intervention (TEACH, 6 sessions with caregivers)	No control group	PROMIS fatigue subscale	After intervention	≤17 years (n = 10): Mean T1 = 20.00, Mean T2 = 14.60, Average decrease = 5.40; ≥18 years (n = 4): Mean T1 = 30.00, Mean T2 = 16.25, Average decrease = 13.75	Significant improvement in fatigue
Fuchs et al., (2013) [19]	Netherlands	Controlled trial	42	Adolescents with JIA	Self-confrontation psychological intervention	Usual care	CIS-20	After 11 sessions	No differences in fatigue between the 2 intervention groups	Psychological intervention reduces fatigue
Hilderson et al., (2016) [20]	Belgium	Quasi-experimental group design	Not reported	Adolescents with JIA	Transition program for transfer to adult rheumatology	Usual care	MFI-20	After transition	Small positive effects: Reduction in mental fatigue (ES = 0.28), general fatigue (ES = 0.27), and physical fatigue (ES = 0.22); Increased activity (ES = 0.27) and motivation (ES = 0.23)	The transition program reduces fatigue

Interventions varied across studies and included exercise-based programs such as resistance and aerobic training, aquatic-based exercises, and cognitive-behavioral interventions. Additionally, pharmacological and nutritional interventions, including prednisolone treatment, vitamin D supplementation, and calcium supplementation, were examined. The comparators included usual care, placebo, or no control group, depending on the study design.

Fatigue assessment tools used across studies included the Pediatric Quality of Life Multidimensional Fatigue Scale (PedsQL-MFS) [11,12,14,16,17], Fatigue Severity Scale (K-FSS) [13], 10 cm Fatigue Scale [15], PROMIS (Patient-Reported Outcomes Measurement Information System) Fatigue Subscale [18], Checklist Individual Strength (CIS-20) [19], and Multidimensional Fatigue Inventory (MFI-20) [20]. The timing of outcome assessments varied, with some studies measuring fatigue immediately post-intervention and others conducting follow-ups ranging from weeks to months.

The findings across studies indicated mixed results. Some interventions, such as aquatic-based exercises, CBT, and transition programs, demonstrated significant fatigue reduction, while others, including resistance training and calcium supplementation, did not show statistically significant effects. The overall effectiveness of fatigue-reducing interventions varied depending on the intervention type, duration, and study design.

Results of Risk of Bias Assessment

**Figure 2:**
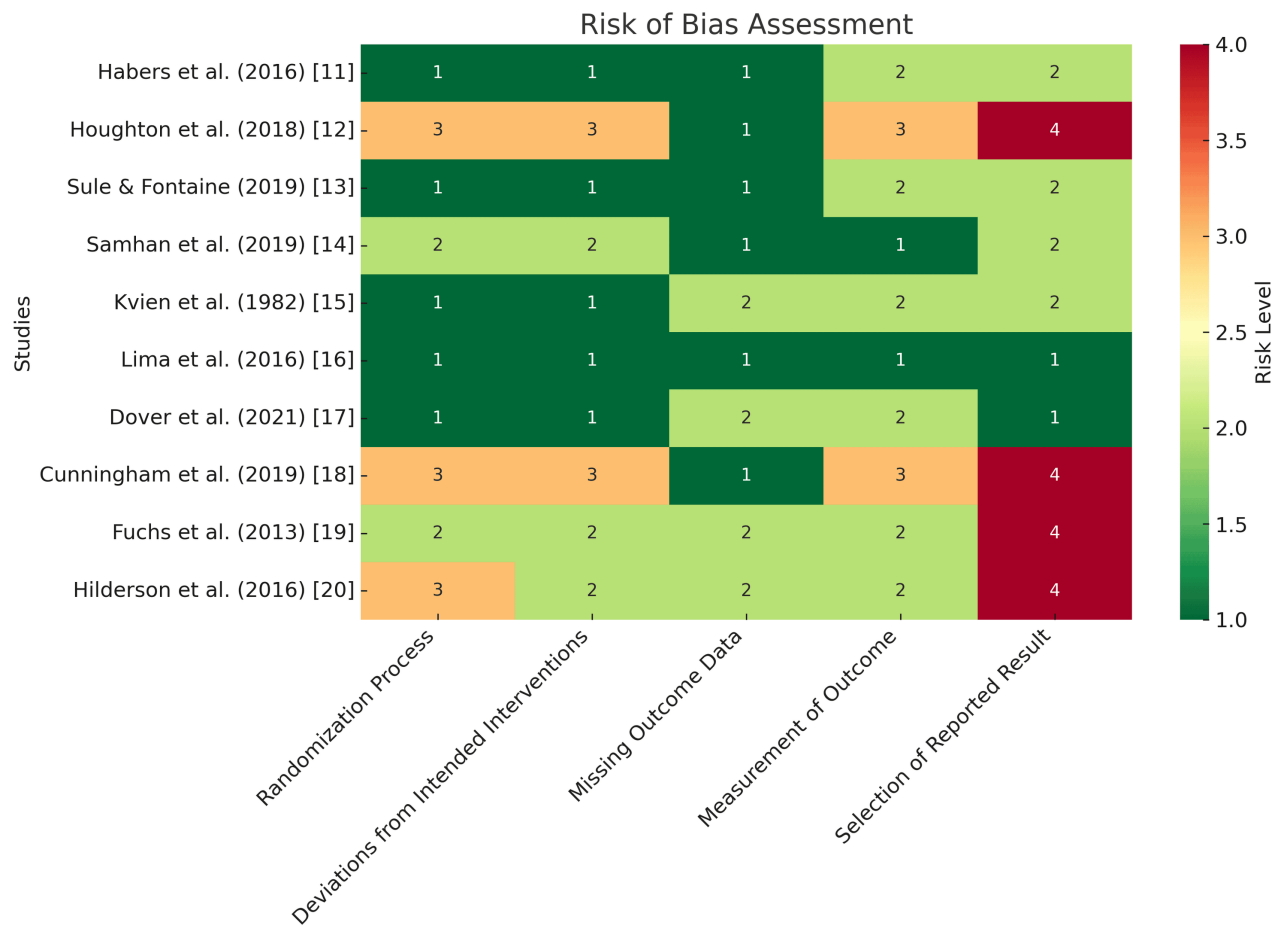
Risk of Bias Assessment of the Included Studies Using the Cochrane Risk of Bias 2 (RoB 2) Tool The five evaluated domains are represented along the x-axis, while the studies are listed on the y-axis. Color coding indicates the level of bias: green (low risk), yellow (some concerns), orange (moderate risk), and red (high risk). Higher bias levels in measurement and reporting domains suggest potential methodological limitations affecting study reliability.

Among the 10 included studies, five were RCTs, while the remaining studies followed controlled or quasi-experimental designs. The risk of bias was generally low in the randomization process, but increased in areas related to measurement and selective reporting. Studies utilizing subjective fatigue scales showed some concerns in outcome measurement due to potential reporting bias. Additionally, trials without robust blinding methods exhibited a higher risk of bias in deviation from intended interventions. Overall, the assessment highlighted methodological limitations that could impact the reliability of reported fatigue outcomes (Figure 2).

Discussion

In children and adolescents with PRCs, fatigue is a common unpleasant condition that can significantly affect the patient's and his or her family's engagement in everyday life and general well-being [11-13]. By keeping fatigue from becoming chronic, early and appropriate assessment, treatment, and reduction of its intensity can enhance both their present and future well-being and engagement in day-to-day activities [18].

Although fatigue has a significant impact and long-term effects on patients with PRC, there are currently few available treatment alternatives [19]. Although the data syntheses show insufficient evidence to support the effectiveness of current interventions to reduce the extent of fatigue in PRCs, the remarkably small number of studies with regard to the effectiveness of interventions aimed at minimizing fatigue among individuals with PRCs suggested a minor yet important impact in the individual studies [11,18]. Fatigue was not the main outcome in the majority of the trials, and none of them reported that the research group was well-controlled with generally low disease activity or adjusted for illness activity. The inconclusive results of the included studies, the risk of bias, variation in diagnostic groups, ages and disease phases, the heterogeneity in the variety of interventions, and the small number of studies all combine to offer a general assessment of the effectiveness of the used interventions, particularly with regard to the potential superiority of one form of therapy [21]. Our findings highlight the need for more multifaceted intervention research primarily focused on treating fatigue in adolescents and children with PRCs, as well as the need for greater understanding of how to measure fatigue in this patient population precisely and consistently throughout treatment and follow-up [22].

The most promising therapeutic modalities for lowering fatigue in individuals with rheumatoid arthritis are physical and psychological therapy [13]. Based on a systematic review of six studies (388 participants) examining physical therapy (such as yoga, strength training, aerobics, and pool-based therapy) and 13 studies (1579 participants) examining psychological approaches (such as self-management, CBT, mindfulness, and group education), Kim et al. [23] concluded that both psychological interventions and physical activity had a marginally significant impact on reducing fatigue in adults with RA. Hewlett et al. [24] recently reported that group CBT had a positive influence on lowering the impact of fatigue in rheumatoid arthritis patients when compared to just obtaining fatigue information. Although further research is necessary, CBT may potentially be useful for people with PRCs.

Similar to this study, all of the previously stated reviews note that the studies that were included had heterogeneous interventions, small sample numbers, and varying outcome measures and intervention protocols, which means they do not produce definitive proof [25,26].

This review's strength is its emphasis on strategies to address fatigue in PRC patients. However, the data presented by the studies that satisfied the inclusion criteria did not allow for meta-analysis. Few, varied, and inconclusive studies constitute the small number of included studies. Moreover, stratified assessment was not feasible due to the small number of papers available for synthesis and the significant variety in intervention techniques, study designs, and outcome-measuring tools among the individual research. As a result, it is impossible to offer suggestions for incorporating successful interventions into children with PRCs' everyday routines [27].

In addition to being a medical (side) effect of PRCs and their treatment, fatigue is most likely the complicated outcome of the psychological and physical difficulties associated with having a chronic illness growing up [16]. Thus, it may be necessary to look for a multifaceted cause and a dynamic interaction between the body and mind to find the solution. All of the demonstrated therapies targeted distinct (single) potential reasons why adolescents and children with various PRC types might feel exhausted [17]. The conceptual model of fatigue in JIA put forth by Kim et al. [23] shows that fatigue appears to be multifactorial and ought to be handled as such. The model depicts a potential interaction between "personal," "disease-related," "environmental," and "generic" elements that may be involved in the development and persistence of fatigue in PRCs. As a result, we recommend that treatments, including CBT, address both the psychological and physical components of weariness [17]. It has been demonstrated that CBT helps fatigued adults with a variety of chronic illnesses, including RA, as well as adolescents with chronic fatigue disorders [14]. Additional promising therapies include calm, healing touch, exercise, and education. Through lowering inflammation, boosting muscular growth or strength, and enhancing mental and functional abilities, physical activity may help reduce weariness.

Limitations

This review is subject to several limitations. The small number of included studies (n=10) and the heterogeneity of the interventions (e.g., CBT, physical therapy), outcome measures, and patient populations (e.g., varying diagnoses and age groups) prevented the conduct of a meta-analysis and stratified assessments, as discussed. Additionally, most studies had methodological flaws, including a high risk of bias in randomization and attrition (Figure 2), and fatigue was often assessed as a secondary outcome, which resulted in underpowered analyses. The use of inconsistent fatigue measurement tools, the absence of long-term follow-up (e.g., Lima et al. [16]), and the potential for publication bias further limit the strength of the conclusions. While these limitations reflect the early stage of fatigue intervention research in PRDs, as discussed, they emphasize the urgent need for larger, well-designed, and standardized trials that prioritize fatigue as a primary endpoint.

## Conclusions

More intervention studies focusing only on treating fatigue in adolescents and children with PRCs are desperately needed. Potential underlying biological and psychological pathways must be identified as potential therapy targets to lessen fatigue symptoms in kids and teenagers with PRCs. Future research should look into interventions that use a multifaceted approach to fatigue. This means that they should focus on the psychological and physical aspects of fatigue in addition to an evaluation that determines how fatigue relates to environmental, personal, and physical outcome parameters.
